# Epidemiology and Evolution of Emerging Porcine Circovirus-like Viruses in Pigs with Hemorrhagic Dysentery and Diarrhea Symptoms in Central China from 2018 to 2021

**DOI:** 10.3390/v13112282

**Published:** 2021-11-15

**Authors:** Kankan Yang, Menghuan Zhang, Qi Liu, Yingli Cao, Wuyin Zhang, Yueqiao Liang, Xiangjun Song, Kaiyuan Ji, Ying Shao, Kezong Qi, Jian Tu

**Affiliations:** 1 Anhui Province Engineering Laboratory for Animal Food Quality and Bio-Safety, College of Animal Science and Technology, Anhui Agricultural University, Hefei 230036, China; ykk@stu.ahau.edu.cn (K.Y.); 18317560317@stu.ahau.edu.cn (M.Z.); 20720428@stu.ahau.edu.cn (Q.L.); cyl@stu.ahau.edu.cn (Y.C.); 123@stu.ahau.edu.cn (W.Z.); lyq@stu.ahau.edu.cn (Y.L.); sxj@ahau.edu.cn (X.S.); 18234487253@163.com (K.J.); shaoying@ahau.edu.cn (Y.S.); 2 Anhui Province Key Laboratory of Veterinary Pathobiology and Disease Control, Anhui Agricultural University, Hefei 230036, China

**Keywords:** porcine circovirus-like virus, pregnant sows, recombination, selection pressure, central China

## Abstract

Porcine circovirus-like virus (PCLV) is a type of circular *Rep*-encoding single-stranded DNA virus and may be associated with the development of diarrheal symptoms in pigs. In this study, we retrospectively analyzed three years of past cases in Anhui, China, and reported a case of hemorrhagic enteritis and death in a pregnant sow possibly caused by PCLV. In addition, we analyzed the evolutionary characteristics of PCLV and found that mutation, recombination and selective pressure all played an important role in the evolution of PCLV. We identified N15D and T17S as well as L56T, T58R, K59Q, M62R, L75I and R190K mutations in two different branches, and we noted recombination events in the *Rep* of a group of Chinese strains. Analysis of selection pressure revealed that PCLV gained more positive selection, indicating that the virus is in a continuous evolutionary state. The PR2 plot, ENC-plot and neutrality analysis showed a greater role of natural selection than that of mutational pressure in the formation of codon usage patterns. This study is the first to identify PCLV in sows with hemorrhagic dysentery and death, and it provides new epidemiological information on PCLV infection in pigs in China.

## 1. Introduction

Circular *Rep*-encoding single-stranded DNA (CRESS DNA) viruses form a highly diverse group of small viruses that have been found worldwide in prokaryotic, eukaryotic and even environmental samples [1,2]. The first eukaryotic CRESS DNA viruses were not discovered until the 1970s, although symptoms consistent with CRESS DNA virus infections were described more than a thousand years ago in plants [3]. Currently, CRESS DNA viruses are divided into seven families: *Bacilladnaviridae*, *Nanoviridae*, *Smacoviridae*, *Geminiviridae*, *Genomoviridae*, *Redondoviridae* and *Circoviridae* [4]. Members of the *Circoviridae*, *Smacoviridae* and *Redondoviridae* families are known to infect animals, including mosquitoes, rats, bats, ducks, cattle, pigs, dogs, humans, turkeys, and forest musk deer [5,6,7,8]. In addition, a new family, *Kirkoviridae*, was proposed by The International Committee on Taxonomy of Viruses (ICTV). The genome structure of members of this family contains only one open reading frame (ORF), encoding only the *Rep*, rather than containing two ORFs as in the case of members of other families [2,9,10].

Porcine circovirus-like virus (PCLV) is a closed circular, non-enveloped, single-stranded DNA virus, similar to porcine circovirus (PCV), and it was identified as a member of the family *Kirkoviridae*. As mentioned above, PCLV, containing only one ORF, is significantly different from PCV in gene structure [11]. The virus was discovered in fecal samples in the United States in 2011 [12]. In 2020 and 2021, the virus was found in Guang-xi and Guangdong provinces, located in the southern part of China [13,14]. At present, most studies show that the virus is found in piglets and is less likely to be reported in adult pigs; it causes hemorrhagic enteritis in piglets, seriously compromising their health [12,14]. In addition to causing clinical signs in piglets, the virus can also cause co-infections with other viruses, such as porcine circovirus 2 (PCV-2) and porcine epidemic diarrhea virus (PEDV), making prevention and control of the virus infection challenging.

Based on this, 140 diarrheal and non-diarrheal clinical samples (including lung, intestine, spleen, fecal, etc.) were collected from 13 pig farms in five cities in Anhui Province, China, spanning from June 2018 to August 2021. The samples were retrospectively investigated in this study to evaluate and enrich knowledge of the epidemiological characteristics and genetic diversity of PCLV. An unexplained case of sow diarrhea and death on a pig farm in Anhui Province on 15 July 2021 was also analyzed. Polymerase chain reaction (PCR) was used to monitor the prevalence of PCLV in central China, to amplify the full genome length of three strains, and to analyze the homology and evolutionary characteristics between different PCLV genomes. In this study, basic information on PCLV is provided to facilitate further understanding of its characteristics; however, its clinical significance, epidemiology and disease prevention deserve further in-depth study.

## 2. Materials and Methods

### 2.1. Sample Collection

To investigate the molecular genetic diversity and epidemiological characteristics of PCLV circulating in Anhui Province, China, and whether they correlate with signs of diarrhea, a total of 140 clinical samples from diarrheic and non-diarrheic adult pigs and piglets were collected from thirteen pig farms in Anhui Province from June 2018 to August 2021. All samples were transported packed on ice and stored at −80 °C.

### 2.2. Viral Nucleic Acid Extraction and PCR Detection

Fecal material (2 g) was dissolved in an Eppendorf tube containing 10% phosphate-buffered saline (PBS) and clarified by centrifugation for 3 min at 12,000× *g*. Tissue samples were added to PBS at a ratio of 1:3 and homogenized in a mortar, followed by centrifugation for 5 min at 13,000× *g*. Following this, 200 µL of supernatant was transferred to sterilized 2 mL centrifuge tubes, and viral DNA was extracted using the TIANamp Virus DNA/RNA Kit (Tiangen, Beijing, China), according to the manufacturer’s instructions. The known genomic sequence of PCLV was retrieved from Genbank and analyzed using the Lasergene package (DNAStar Inc., Madison, WI, USA). A pair of detection primers was designed for the conserved region (Table S1), and PCR was performed under the following conditions: an initial denaturation step at 94 °C for 5 min; 35 cycles at 94 °C for 30 s, at 49 °C for 30 s, and at 72 °C for 30 s; and finally extension at 72 °C for 5 min. The PCR products were analyzed by agarose gel electrophoresis, and the target bands were cut off for DNA extraction using a gel extraction kit and sent to TsingKe Co., Ltd. (Nanjing, China), China for sequencing before blast search verification.

### 2.3. PCLV Sequencing of the Full-Length Genome

**Figure 1:**
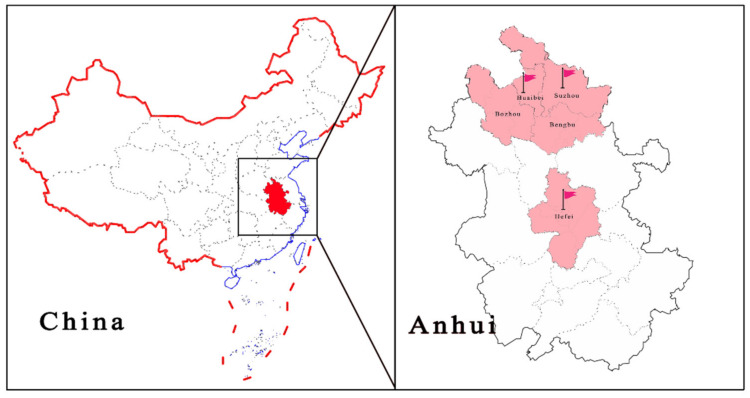
Geographic distribution of positive samples.

Full-length primers were designed using the primers in previously published studies and for the reference strain (accession number: NC_025682.1) to amplify the genomes of samples that tested positive for PCLV. The primers are shown in Table S1. The geographical distribution of positive samples was marked on a map (Figure 1). All amplification products were visualized by agarose gel electrophoresis and then purified using a DNA purification kit (TIANGEN, Beijing, China) according to the manufacturer’s instructions. The PCR products were cloned into the pMD19-T vector (TaKaRa, Kusatsu, Japan), and the recombinant plasmids were transformed into Trelief™ 5α chemically competent cells (TsingKe, Nanjing, China), extracted using a QIAGEN Plasmid Mini Kit, and sent to TsingKe Co., Ltd. (Nanjing, China) for bidirectional sequencing. The reference strains were obtained from the NCBI database (https://www.ncbi.nlm.nih.gov/nucleotide/; accessed on 2 September 2021 (Table S3). Multiple sequence alignments were constructed using Clustal W.

### 2.4. Sequence Alignment, Phylogenetic and Recombination Analysis

The reference strains were sourced from the NCBI database, as shown in Table S3. Sequence identity was analyzed using Megalign (DNASTAR, Madison, WI, USA). Sequence alignment was performed based on nucleotide sequences via MAFFT (https://mafft.cbrc.jp/alignment/software/, accessed on 29 August 2021) and ESPript Software (https://espript.ibcp.fr/ESPript/ESPript/; accessed on 2 September 2021). Phylogenetic analysis was performed using PhyloSuite software [15]. Maximum likelihood phylogenies were inferred using IQ-TREE for 5000 ultrafast bootstraps and the Shimodaira–Hasegawa-like approximate likelihood-ratio test [16,17]. ModelFinder was used to select the best substitution model [18]. Phylogenetic trees were visualized using FigTree software.

Recombination events were identified using seven methods in the Recombination Detection Program v.4.39 (RDP 4.39), namely, *RDP*, *GENECONV*, *Chimaera*, *MaxChi*, *BootScan*, *SiScan* and *3Seq.* SimPlot software v.3.5.1 was used to validate and visualize reorganization events.

### 2.5. Selection Pressure, B-Cell Epitope Prediction and Codon Usage Bias Analyses

Analysis of the selection pressure on *Rep* was performed using four methods, namely, Single-Likelihood Ancestor Counting (SLAC), Fixed Effects Likelihood (FEL), Fast Unconstrained Bayesian AppRoximation for inferring selection (FUBAR), and Mixed Effects Model of Evolution (MEME) on DATAMONKEY (http://www.datamonkey.org/; accessed on 20 August 2021) [19,20,21]. Significant recombinant strains were excluded. The B-cell epitope was predicted via SVMTriP (http://sysbio.unl.edu/SVMTriP/ (accessed on 10 August 2021) [22]. Protein structures were visualized using PyMol v4.60.

Analysis of codon usage bias was performed online using the CAIcal SERVER (https://ppuigbo.me/programs/CAIcal/; accessed on 15 August 2021). The host’s (*Sus scrofa*) codon usage pattern was obtained from the codon usage database (http://www.kazusa.or.jp/codon/; accessed on 26 August 2021). A Parity Rule 2 (PR2) plot was used to analyze whether the effects of mutation pressure and natural selection on the use of the *Rep* codon were consistent. In a PR2 plot, [A3/(A3 + T3)] is the ordinate and [G3/(G3 + C3)] is the abscissa. The center of the plot shows A = T and G = C, indicating that there is no deviation between mutation pressure and the influence of natural selection [23,24,25].

The value of ENC ranged from 20 to 60, negatively correlating with codon usage bias. ENC ≤ 35 indicates high codon usage bias, and ENC > 50 indicates that the codon usage is low [26]. The ENC-GC3s plot (ENC-plot) was analyzed with the ENC values plotted against the GC3S values to determine the main factors affecting codon use bias. The expected ENC values for each GC3S were estimated as follows [26]:

(1) $$y=2+x+\frac{29}{x^{2}+{\left(1-x\right)}^{2}}$$

where *x* represents the value of G + C at the third codon position (GC3S).

Neutrality analysis was used to evaluate the effects of mutation pressure and natural selection on codon usage patterns. With GC12 as ordinate and GC3 as abscissa, the neutrality plot was generated. If the slope of the regression line was close to 1 (the point on the diagonal), there was no or weak selection pressure. In addition, the deviation from the diagonal of the regression line indicated the magnitude of the influence of natural selection [27]. Data visualization was completed using Origin 2018.

## 3. Results

### 3.1. Prevalence of PCLV

A total of 140 samples collected from different regions (Suzhou, Huaibei, Bozhou, Bengbu and Hefei) of Anhui province were tested, and three positive samples, collected in the years 2018, 2019 and 2021 from three different pig farms, A, B and C, were found (Figure 1), with a positive rate of 2.14% (3/140). As mixed infections of pathogens are common in clinically affected pigs, we investigated the mixed infections in three samples. Sample 1 (AH-23, accession no. MZ773067) from farm A was a fecal sample from a weaned piglet that showed signs of diarrhea, and PCLV was detected mixed with porcine astrovirus and PEDV. The age of the pig from which sample 2 was collected (AH-25, accession number: MZ773068) on farm B could not be determined, and the fecal sample was co-infected with PCV-2 and porcine parvovirus (PPV). Notably, sample 3 (AH-HB-2021, accession no. MZ960935) from farm C was collected from a pregnant sow with hemorrhagic enteritis, which eventually led to the death of the sow (Figure S1), and eight sows from the batch showed signs of diarrhea, with four cases resulting in death. Sample 3 was negative for other viruses routinely infecting pigs (African swine fever virus (ASFV), pseudorabies virus (PRV), porcine circovirus 2 (PCV-2), porcine circovirus 3 (PCV-3), porcine reproductive and respiratory syndrome virus (PRRSV), porcine epidemic diarrhea virus (PEDV), transmissible gastroenteritis virus (TGEV), porcine parvovirus (PPV), porcine rotavirus A (PRV-A) and classical swine fever virus (CSFV), among others) using commercially available kits, and primer sequences for pathogens (porcine astrovirus (PAstV), porcine kobuvirus (PKV), porcine deltacoronavirus (PDCoV), porcine bufavirus (PBuVs) and porcine sapovirus (PSaV)) without commercially available kits are shown in Table S2.

### 3.2. Full-Sequence Alignment of PCLV

**Figure 2:**
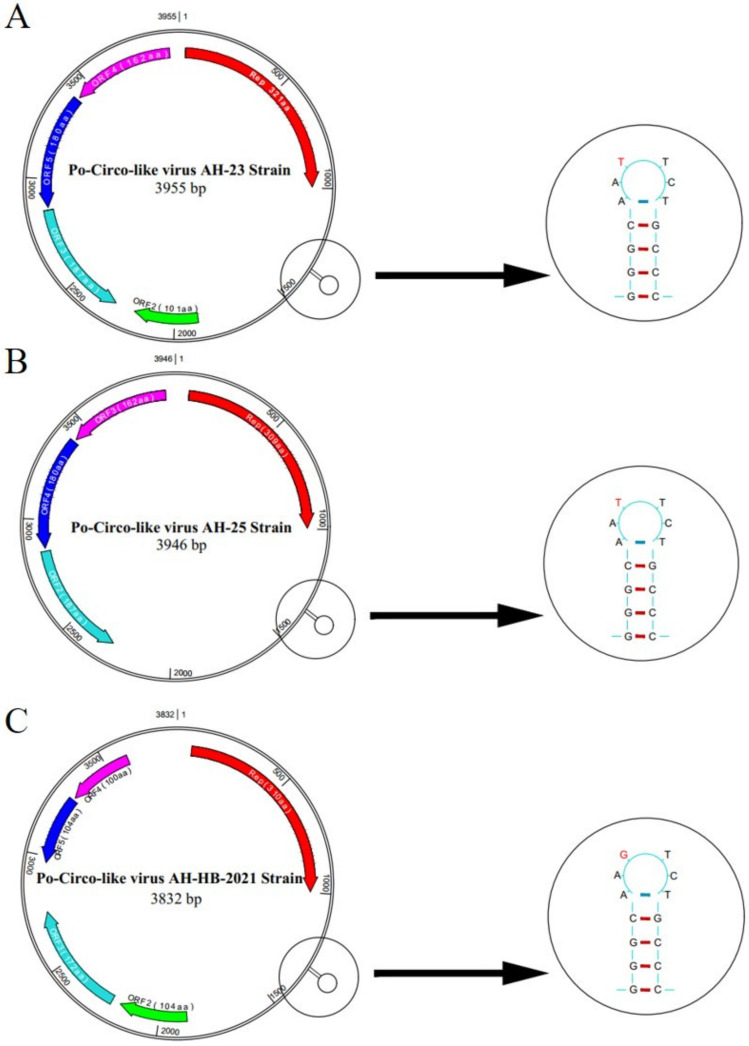
Predicted genomic structural characteristics of PCLV-AH-23, PCLV-AH-25 and PCLV-AH-HB-2021. PCLV-AH-25 only four potential ORFs were present, whereas in strain PCLV-AH-23 and PCLV-AH-HB-2021, the locations of five potential ORFs were identified,, one of which contained the opposite transcriptional direction. A significant T7G mutation was found in the stem loop of PCLV-AH-HB-2021.

**Figure 3:**
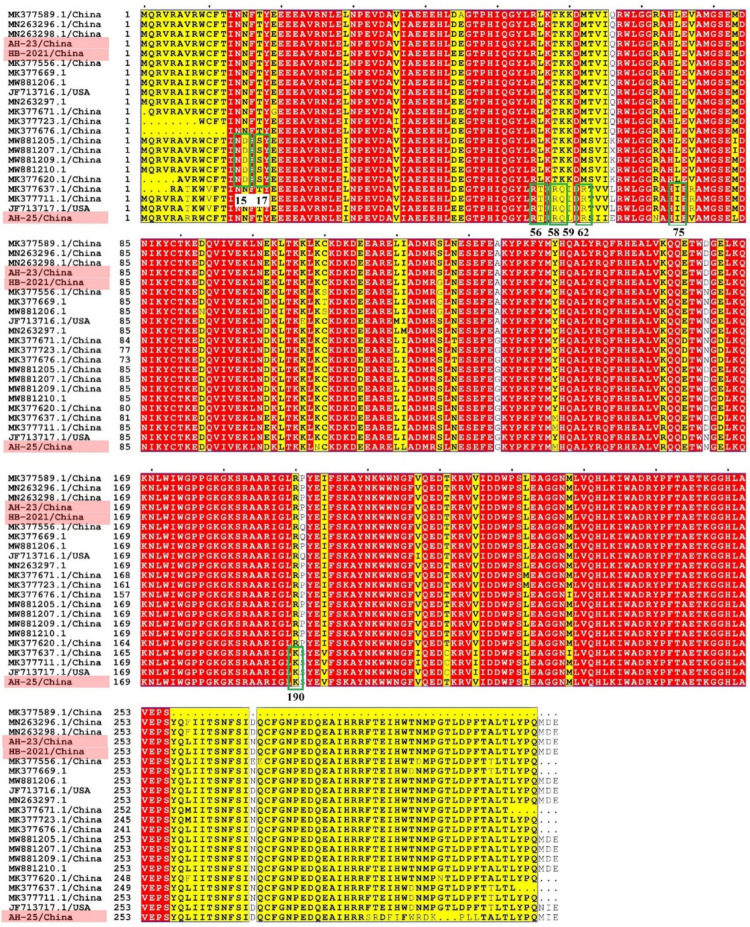
Amino acid mutation analysis of the PCLV *Rep*. Strains obtained in this study are indicated in pink. The conserved regions of the amino acid sequences are covered in red.

**Figure 4:**
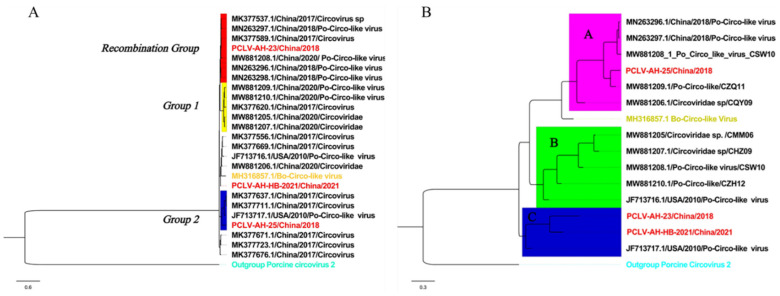
Phylogenetic analysis based on complete sequences and *Rep*. Strains from this study are marked in red. (**A**) *Rep*: The best substitution model was TIM2 + F + G4. Branches marked in yellow are those with recombination events, and Mutation Groups 1 and 2 are strains with mutations. (**B**) Complete sequences: The best substitution model was TPM3 + F + G4. The tree is divided into three branches, each underlined with a different color.

We successfully amplified the full length of three PCLV strains, which had nucleotide lengths of 3955, 3946 and 3832 bp, respectively. In AH-23 and AH-HB-2021, five potential ORFs were identified, while only four potential ORFs existed in AH-25 (Figure 2). Sequence alignment showed that the similarity between the three strains obtained in this study was 86.8~91.1%, and the similarity between the three strains and reference strains was 77.7~94.6%. The nucleotide similarity and amino acid similarity of *Rep* among all strains were 83.4~97.5% and 84.7~99.6%, respectively. Analysis of amino acid mutations revealed that most amino acids in *Rep* were relatively consistent, with a small number of non-synonymous substitutions, while there were relatively more mutations in the *C*-terminus of *Rep*. Notably, *N15D* and *T17S* mutations were found in five strains. In addition, a wider range of consistent mutations was found in four other strains. The mutation sites were *L56T*, *T58R*, *K59Q*, *M62R*, *L75I* and *R190K* (Figure 3). Interestingly, from the phylogenetic tree for the *Rep* (Figure 4A), we found that Group 1 had mutations of *N15D* and *T17S* as mentioned above, while Group 2 had mutations of *L56T, T58R, K59Q, M62R, L75I and R190K*.

### 3.3. Phylogenetic Analysis and Genetic Divergence

**Figure 5:**
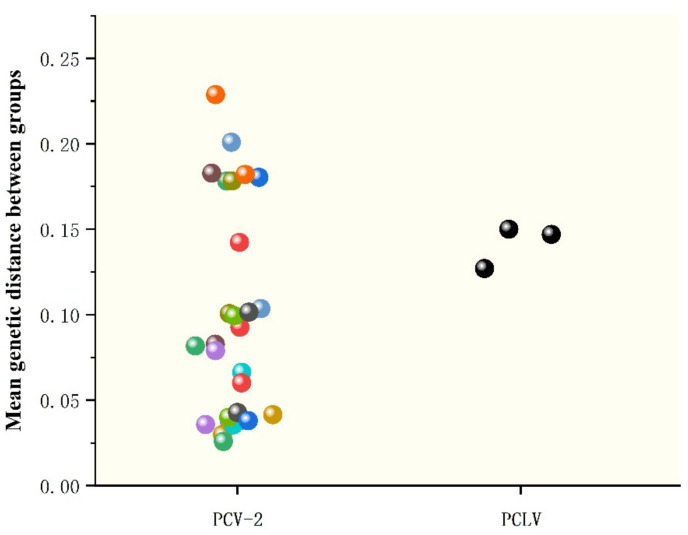
Genetic distance analysis between PCLV and PCV2. The genotypes of PCV refer to the classification of Marina Sibila et al. [28], including 2a, 2b, 2c, 2d, 2e, 2f, 2g and 2h. Genetic distance was calculated using MEGA X with a p-distance model and 1000 bootstrap replicates.

Phylogenetic analysis based on complete sequences showed that all strains were divided into three groups, tentatively named Groups A, B and C. Among them, strains AH-23 and AH-HB-2021 were in a branch with the Po-Circo-like virus 22 strain found in the United States in 2010, while AH-25 was closer to the Po-Circo-like virus isolate CZQ11 isolated in China (Figure 4B). We compared the average between-group genetic distance for each genotype of PCV-2, and the results showed that the genetic distance between the three groups of PCLV was similar to that between PCV-2 types 2e and 2d, less than for 2a–2b, 2a–2c, 2a–2d, 2a-2e, 2a-2f and 2a-2g, and larger than that between other genotypes (Figure 5).

### 3.4. Recombination, Selection Pressure and B-Cell Antigenic Epitope Analysis

**Figure 6:**
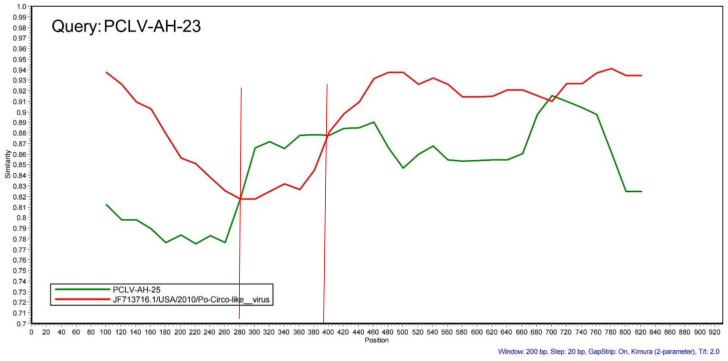
Recombination analysis of PCLV. PCLV-AH-23 was the query sequence, and the major and minor parents were strain PCLV-AH-25 (accession number: MZ773068/China) and Po-Circo-like virus 21 (accession number: JF713716.1/USA). The red curve represents the similarity of strain Po-Circo-like virus 21 to strain PCLV-AH-23. The green curve represents the similarity of strain PCLV-AH-25 to strain PCLV-AH-23. *Rep* is the potential recombination region. Recombination events were identified using seven methods in the Recombination Detection Program v.4.39 (RDP 4.39), namely, RDP, GENECONV, Chimaera, MaxChi, BootScan, SiScan and 3Seq. The SimPlot software v.3.5.1 was used for the analysis with visual similarity plots using a window size of 200 nucleotides that was moved along in 20 nt steps.

**Figure 7:**
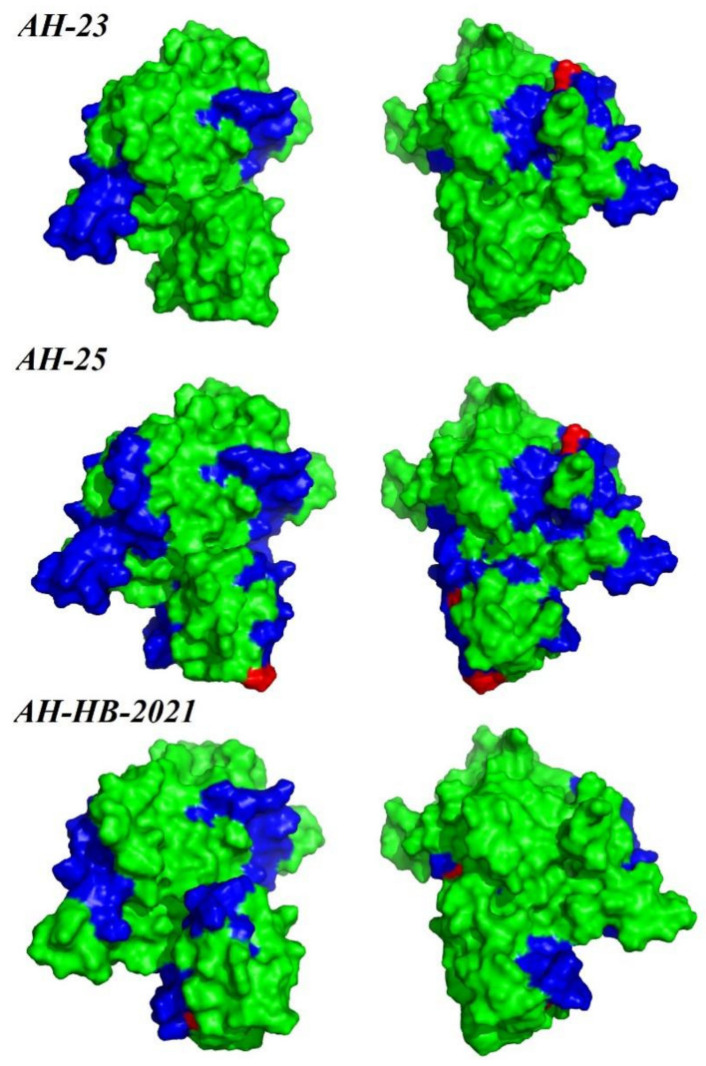
Map of B-cell epitope prediction and pressure selection sites of *Rep*. The predicted epitopes in the *Rep* protein are indicated in blue. The selective pressure sites are indicated in red. Protein structure visualization was performed using PyMol.

In the recombination analysis, extensive recombination events in *Rep* were found in a group of Chinese strains (*Recombination Group*, Figure 4A and Figure 6), and these were detected by four different methods (*MaxChi*, *Chimaera*, *SiScan*, *3Seq*). The represented major parent strain and the minor parent strain were strain PCLV-AH-25 (accession number: MZ773068/China) and strain 21 (accession number: JF713716.1/USA), respectively. Analysis of selection pressure showed that PCLV was subject to positive selection (Table 1), which may lead to the tendency of the virus to mutate and the continuous evolution of the virus, possibly prompting the emergence of new varieties [29,30]. In order to explore whether the selection would affect the immunogenicity of *Rep*, the B-cell epitopes were predicted (Table S4), and the results showed that two positive selection sites overlapped with immune epitopes in AH-23 (codons 142 and 211), six in AH-25 (codons 29, 142, 81, 211, 290 and 72) and two in AH-HB-2021(codons 47 and 211) (Figure 7).

### 3.5. Codon Usage Bias Analysis

**Figure 8:**
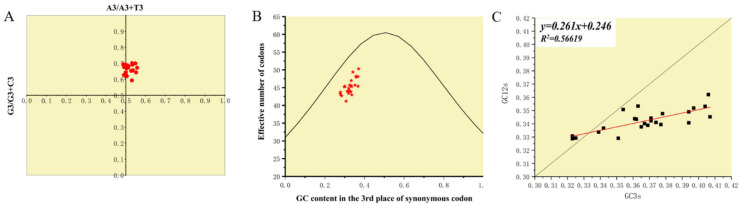
Analysis of factors mediating codon usage bias. (**A**) Parity Rule 2 (PR2) plot was used to analyze whether the effects of mutation pressure and natural selection on the use of the *Rep* codon were consistent. Figure 8A shows that mutation pressure and natural selection have unequal effects on the use of codons during evolution. (**B**) The ENC-GC3s plot was analyzed, with the ENC values plotted against the GC3S values to determine the main factors affecting codon use bias. The expected ENC values for each GC3S were estimated. Figure 8B shows that main factor affecting codon use bias was selection pressure. (**C**) Neutrality analysis was used to evaluate the effects of mutation pressure and natural selection on codon usage patterns. Figure 8C shows that the fitting curve (red line) deviates from the curve *y = x* (black line), indicating that selection pressure plays a dominant role, which is consistent with Figure 8B.

The ENC average of PCLV was 45.296 (41.2~50.3), indicating that PCLV has a low codon bias. PR2 plot analysis found that no strains clustered in the center of the coordinate axis (Figure 8A), indicating that the influence of mutation pressure and selection pressure on codon bias was inconsistent. To explore which factor was more biased towards codons, ENC-plot analysis was performed (Figure 8B). The results showed that all strains were below the expected curve, indicating that selection pressure plays a more important role in *Rep* during evolution. Neutrality analysis also suggested that natural selection had a greater effect on codon bias than mutation pressure (Figure 8C); the contribution of natural selection was about 73.9%.

## 4. Discussion

Circular *Rep*-encoding (replication-associated protein encoding) single-stranded DNA (CRESS DNA) viruses are a major component of the earth virome and can be detected and show high diversity and abundance in prokaryotic and eukaryotic organisms as well as in environmental samples worldwide. Currently, a new CRESS DNA virus similar to PCVs, with a circular genome but without the typical capsid protein (*Cap*), has been confirmed in diarrheic pigs. The epidemiology of PCLV in China needs further investigation. In this study, we performed an in-depth analysis and study of the genomic characteristics and genetic diversity of PCLV in Anhui Province, central China. PCLV infection in piglets has been confirmed in a previous report [13]. However, the results of this study suggest that PCLV may not only infect piglets, causing diarrhea, but may also be able to infect sows, causing severe diarrhea and mortality. 

*Rep* is a relatively conserved gene in ssDNA viruses [2], which was verified in this study. Potential functional analysis of the ORFs revealed that a helicase domain (IPR027417, SSF52540, G3DSA:3.40.50.300) was found in *ORF1 (Rep)*, whereas no domain was found in other ORFs. These domains are closely related to virus replication, and similar domains were also found in PCV [31], again confirming the function of ORF1 in encoding replicase. In addition, we found multiple amino acid mutations by complete genome sequence alignment. These mutations may be the underlying cause of these strains forming separate clades and may be key mutations that promote genotypic differentiation. Therefore, more serious consequences should be anticipated. In PCV, mutation in the *Rep* affect the virus’s ability to replicate, which can increase or decrease depending on the mutation site [32,33,34]. In some viruses, mutations also cause unpredictable effects, such as increased drug resistance, vaccine failure and changing abilities of replication and transmission [34,35,36,37]. Common mutational events in different branches of PCLV may indicate that the hazard of the virus to pigs may be more serious and unpredictable.

Currently, there is no standard and specific classification method for PCLV. In *circoviruses*, the *Cap* is considered to be very important for classification based on the phylogenetic tree and genetic distance [6,38,39]. However, *Cap* cannot be identified in PCLV [27], so the phylogenetic tree structure and genetic distances were investigated in this study. However, whether these three groups can be considered as three genotypes is currently lacking strong evidence, including a suitable genotyping basis and a large number of reference strains. At present, genotypic differentiation is known to be common in members of the genus *Circovirus*, such as PCV-2, PCV-3, duck circovirus and canine circovirus [40]. Given the close relationship between PCLV and PCV-2, more data are needed to prove whether the differentiation of three groups in PCLV also represents different pathogenicity and epidemic potential.

The topological differences between the tree based on *Rep* and the complete genome mentioned above indicate the occurrence of recombination events [41]. *Rep* is considered to be essential for viral replication and plays a crucial role in cellular immunity [6,42]. Recombination events are also widely found in other viruses, such as porcine circovirus, canine circovirus, pigeon circovirus, duck circovirus and goose circovirus [41,43,44,45,46]. The hotspot of recombination events is *Rep*, but it is still relatively conserved in ssDNA viruses, indicating that recombination may have little influence on the diversification of *Rep*. However, given recombination is a driving force in the evolution of viruses, we need to be concerned about the unpredictable effects of recombination on *Rep*.

Selection pressure can come from a wide range of sources, such as the effects of drugs, the host’s own immune system, or “competitors”. In this study, selection pressure on the virus promotes evolution. Unfortunately, we were unable to determine what brings about the selective effect of the virus, which is often not a single factor. The results of selection pressure and antigenic epitopes suggest that continuous changes in the immunogenicity of the virus may result in the diversity of PCLV and reinfection of domestic pigs in which antibodies have been acquired, causing further epidemics. Although *Rep* is not the main gene determining the immunogenicity of the virus, it is related to humoral immunity [42]. The same phenomenon was also found in PCV, especially PCV-2, which led to the worldwide epidemic and had a negative impact on the swine industry [47,48]. Codon usage bias is a pattern of codon use formed by viruses during evolution and influenced by mutational pressure, natural selection and other factors [25,49]. Low codon bias may promote efficient replication by reducing competition between virus and host during protein synthesis and may increase virulence. The virulence of PCLV may be underestimated currently or may increase in the future due to this low codon bias. Therefore, we should be more vigilant about the harm PCLV causes to the swine industry. In addition, the codon bias is always influenced by external factors to varying degrees. Natural selection has been shown to play a major role in PCV-2 and canine circovirus [50,51], but mutation is more important in duck circovirus codon bias [52]. Although these viruses are closely related, different factors have influenced their evolution. Therefore, as both natural selection and mutation pressure appear to influence the evolution of PCLV, further attention should be paid to these factors. 

In conclusion, PCLV is capable of causing diarrhea not only in piglets but also in pregnant sows and can also cause death, as elucidated in this study. The pathogenicity of PCLV may be enhanced and revealed gradually with further evolution of amino acid mutations. However, further experiments on the construction of PCLV-based infectious clones are needed to elucidate the biological properties and pathogenic mechanisms of the mutation of this virus locus in vivo and in vitro.

## Figures and Tables

**Table 1:** Positive selection site for three genotypes of PCLVs.

Methods	Positive Selection Sites	Threshold
FUBAR	29, 142	*p* < 0.1
FEL	29	
MEME	47, 81, 142, 211, 290, 8, 309, 72, 29	
SLAC	/	Posterior probability of 0.9

## Data Availability

The data set supporting the conclusions of this article is available in GenBank.

## Supplementary Materials

The following are available online at https://www.mdpi.com/article/10.3390/v13112282/s1, Figure S1: Pregnant sows developed diarrhea and hemorrhagic enteritis. Table S1: Primer sequences for detection and amplification of the whole genome of PCLV. Table S2: The primers for porcine virus detection without commercial kits. Table S3: The reference strain information used in this study. Table S4: The results of B-cell epitope prediction.
