# Supplementary material for: Epidemiology and Evolution of Emerging Porcine Circovirus-like Viruses in Pigs with Hemorrhagic Dysentery and Diarrhea Symptoms in Central China from 2018 to 2021
Source: Viruses. 2021 Nov 15;13(11):2282. doi: 10.3390/v13112282 (PMC8624291; doi:10.3390/v13112282)
Supplement: Supplementary file 1 [file viruses-13-02282-s001.zip › viruses-1427538-supplementary.pdf]

Supplementary file Figure S1. Pregnant sows developed diarrhea and hemorrhagic enteritis.

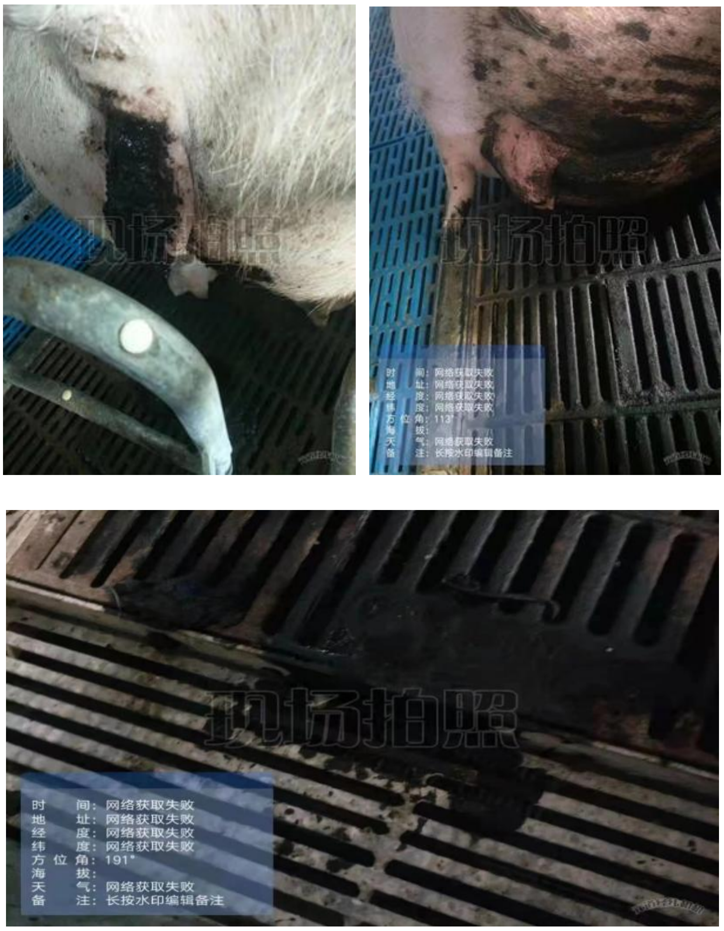

Supplementary file Table S1 Primer sequences for detection and amplification of the whole genome of PCLV .

Isolated strain	Primer name	Primer Sequence (5' to 3')	Size(bp)	Purpose
	PCLV-D-F	TGCTGAGACTAAGGGAGG	439	Detection
	PCLV-D-R	ACTGGGTAAACGTAATGGA		
AH-23	PCLV-1-F	GGTTCCTATTATGGTCCGATTTTTGTGG	1102	Genome sequencing
	PCLV-1-R	CTCCTTGTGCATCTGGGTATTGCTTGTC		
	PCLV-2-F	GACAAGCAATACCCAGATGCACAAGGAG	2853	Genome sequencing
	PCLV-2-R	CCACAAAAATCGGACCATAATAGGAACC		
AH-25	PCLV-3-F	ATGCCTGGCACCTTAGACCCCTTTA	916	Genome sequencing
	PCLV-3-R	AATGAACTGACCACTCATGAA		
	PCLV-4-F	TTCATGAGTGGTCAGTTCATT	3030	Genome sequencing
	PCLV-4-R	TAAAGGGGTCTAAGGTGCCAGGCAT		
AH-HB-2021	PCLV-5-F	TAGGGATTTCCGCTTGGATCAAGTACT	1370	Genome sequencing
	PCLV-5-R	TATTACCTTTAGCGGAATCAAAATCGGAC		
	PCLV-6-F	GTCCGATTTTGATTCCGCTAAAGGTAATA	2462	Genome sequencing
	PCLV-6-R	AGTACTTGATCCAAGCGGAAATCCCTA		

Supplementary file **Table S2** The primers for porcine virus detection without commercial kits

**Virus**	**Primer**	**Sequence**	**Product size （bp）**
PBuVs	PBuVs-F	GGGCGAAGTTATTATCGTAT	102
	PBuVs-R	AATCTGGGTCCTCTGTCTGTCT	
PKoV	PKoV-F	GGTCTTTGGCTACCTTCTTGTTC	342
	PKoV-R	GTTTGTGGGTCAGTCTTCGCTT	
PDCoV	PDCoV-F	ATGCTCAAATCAACGAAACAC	265
	PDCoV-R	TCACCACTATCATCCTCACCC	
PAstV	PAstV-F	ACCACCGCGCAGGA	573
	PAstV-R	TGTTGYTCAAGRGCAG	
PSaV	PSaV-F	CGCTTTCCAATCATCACTCCAGG	177
	PSaV-R	GGTGTTGTCACGGGTGTTCAGGT	

Supplementary file Table S3 The reference strains information used in this study

Strains	Collection date	Origin	Nucleotide(nt)	Genbank accession No.	Host	Organism
**HB-2021**	**2021**	**China**	**3832**	**MZ960935**	**Pig**	**Po-Circo-like virus**
**AH-23**	**2021**	**China**	**3955**	**MZ773067**	**Pig**	**Po-Circo-like virus**
**AH-25**	**2021**	**China**	**3946**	**MZ773068**	**Pig**	**Po-Circo-like virus**
PCLV-21	2011	U.S.A	3912	JF713716.1	Pig	Po-Circo-like virus
PCLV-22	2011	U.S.A	3923	JF713717.1	Pig	Po-Circo-like virus
GX14	2019	China	3944	MN263296.1	Pig	Po-Circo-like virus
GX15	2019	China	3944	MN263298.1	Pig	Po-Circo-like virus
GX19	2019	China	3944	MN263297.1	Pig	Po-Circo-like virus
CSW10	2021	China	3950	MW881208.1	Pig	Po-Circo-like virus
CZQ11	2021	China	3946	MW881209.1	Pig	Po-Circo-like virus
CZH12	2021	China	3954	MW881210.1	Pig	Po-Circo-like virus
CQY09	2020	China	3924	MW881206.1	Pig	Circoviridae sp.
CMM06	2020	China	3943	MW881205.1	Pig	Circoviridae sp.
CHZ09	2020	China	3943	MW881207.1	Pig	Circoviridae sp.
CH	2016	China	3909	MH316857.1	Bovine	Bo-Circo-like virus
GX01_C4	2017	China	921	MK377537.1	Pig	Circovirus sp.
GZ04_C4	2017	China	831	MK377589.1	Pig	Circovirus sp.
HLJ01_C7	2017	China	906	MK377620.1	Pig	Circovirus sp.
GX05_C2	2017	China	921	MK377556.1	Pig	Circovirus sp.
NM02_C1	2017	China	985	MK377669.1	Pig	Circovirus sp.
HuN02_C8	2017	China	900	MK377637.1	Pig	Circovirus sp.
NM02_C3	2017	China	906	MK377671.1	Pig	Circovirus sp.
YN02_C6	2017	China	897	MK377723.1	Pig	Circovirus sp.
NX01_C5	2017	China	900	MK377676.1	Pig	Circovirus sp.
YN01_C2	2017	China	921	MK377711.1	Pig	Circovirus sp.

Supplementary file Table S4 The results of B-cell epitope prediction

Strains	Location	Eptitope residue	Score
AH-23	137-156	PKFYMYHQALYRQFRHEALV	1
	198-217	AYNKWWNGFVQEDTKRVVID	0.848
	110-129	CKDKDEEARELIADMRSLNE	0.786
AH-25	25 - 44	RNLEINPEVDAVIAEEEHLE	1
	72 - 91	NAHIEVAMGSELDNIKYCTK	0.825
	137 - 156	PKFYMMHQALYRQFRHEALV	0.662
	287 - 306	FIFWRDKPLLTALTLYPQMI	0.589
	198 - 217	AYNKWWNGFVQEDTKRVVID	0.567
AH-HB-2021	40 - 59	EEHLEEGTPHIQGYLRLKTK	1
	120 - 139	LIADMRGLNESEFEAKYPKF	0.778
	198 - 217	AYNKWWNGFVQEDTKRVVID	0.511
